# Pathological analysis and antimicrobial susceptibility of *Chryseobacterium balustinum* RTFCP 298 isolated from diseased rainbow trout, *Oncorhynchus mykiss*

**DOI:** 10.1038/s41598-023-40028-5

**Published:** 2023-08-15

**Authors:** Sumanta Kumar Mallik, Richa Pathak, Neetu Shahi, Krishna Kala, Suresh Chandra, Partha Das, Bhupendra Singh, Mohan Singh, Abhay Kumar Giri, Ritesh Shantilal Tandel, Debajit Sarma, Pramod Kumar Pandey

**Affiliations:** 1 ICAR-Directorate of Coldwater Fisheries Research (ICAR-DCFR), Bhimtal, Nainital, Uttarakhand 263136 India; 2 ICAR-Indian Agricultural Research Institute, Gauriakarma, Hazaribagh, Jharkhand 825405 India

**Keywords:** Microbiology, Zoology

## Abstract

In this study, six isolates of *Chryseobacterium balustinum* were characterized from diseased rainbow trout fingerlings. The virulence characteristics, pathogenicity, and antimicrobial susceptibility pattern of these isolates were investigated. The bacterium showed positive results for catalase, cytochrome oxidase, and aesculin hydrolysis, while negative results were obtained for DNase, gelatinase, methyl red, Voges-Proskauer's reaction, Simon citrate, Hydrogen sulphide, and starch hydrolysis. Amino acid metabolism analysis revealed the inability to metabolize arginine, lysine, and ornithine decarboxylase. Molecular characterization (16S rRNA) and phylogenetic analysis revealed the test isolates as *C. balustinum*, closely related to strain WLT (99.85% similarity) and *C. balustinum* P-27 (99.77%). Virulence assay indicated haemolytic activity and biofilm formation by the test bacterium. The challenge test confirmed moderate pathogenicity in rainbow trout and established Koch's postulates. The clinical manifestations of infection included fin erosion, eye and body surface haemorrhage, exophthalmia, and organ liquefaction. The minimum inhibitory concentrations of various antimicrobials ranged from 1 to > 256 µg mL^−1^. The novel synthetic antimicrobial peptides exhibited MICs of 8 to > 256 µg mL^−1^, suggesting a potential control method. These findings suggest that *C. balustinum* is an opportunistic pathogen with moderate pathogenicity in rainbow trout. Further research on the host–pathogen relationship is necessary to understand virulence characteristics and pathogenicity in aquaculture.

## Introduction

The initial establishment of the genus *Chryseobacterium* involved the reclassification of six bacterial taxa previously assigned to the genus *Flavobacterium*, namely *F. balustinum*, *F. indologenes*, *F. gleum, F. meningosepticum, F. indoltheticum*, and *F. scophthalmum*^1^. Recent advancements in molecular biology and biotechnology have contributed to improved taxonomic clarity and identification of novel chryseobacterial species^2^. As part of the taxonomic changes within the family Flavobacteriaceae, two species initially classified under the genus *Chryseobacterium,* namely *C. meningosepticum* and *C. miricola*, were subsequently assigned to a new genus called *Elizabethkingia*^3^. By 2006, the genus *Chryseobacterium* had expanded to encompass 10 species, and presently, it comprises over 60 species^4^. Taxonomically, *Chryseobacterium* is classified under the Phylum- Bacteroidota, Class- Flavobacteriia, Order- Flavobacteriales, Family- *Weeksellaceae,* and Genus- *Chryseobacterium*.

*Chryseobacterium* spp. are associated with diverse infections in both humans and animals. Among these, *C. meningosepticum* is the most commonly implicated species in human infections, causing conditions such as neonatal meningitis, pneumonia, bacteremia, sepsis, and soft tissue infections, primarily affecting immunocompromised patients^5–8^. Other *Chryseobacterium* spp., isolated from various clinical and environmental sources, include *C. indologenes*^9^ and *C. gleum*^1^. In animals, chryseobacteria have been detected in the midgut of mosquitoes, the gut of cockroaches, millipede faeces, penguin guano, gut homogenates of freshwater copepods, bird feathers, cow's milk, raw meats, and chicken^10–20^. The genus *Chryseobacterium* has also been identified as a pathogen in various fish species, with *Chryseobacterium* species being considered potential emerging pathogens in both freshwater and marine fish globally^4,21–25^. *C. balustinum* was initially isolated from halibut scales (*Hippoglossus hippoglossus*) in the Pacific Ocean^26^. In recent years, several *Chryseobacterium* species have been identified from various sources, including teleosts, and have been associated with bacterial gill diseases and systemic hemorrhagic septicemia in turbot, *Scophthalmus maximus* L, pufferfish, rainbow trout (*Oncorhynchus mykiss)*, Atlantic salmon (*Salmo salar)*, and golden mahseer (*Tor putitora)*^21–24,27–29^. The incidence of *Chryseobacterium* infection in animals has been increasing in South Korea, emphasizing the importance of ongoing monitoring and research on this emerging pathogen^20^. Additionally, *Chryseobacterium* species have demonstrated elevated levels of antibiotic resistance, including resistance to last-resort antibiotics, leading to concerns about their clinical implications and potential for zoonotic transmission^30,31^.

In this study, we aimed to investigate the occurrence of *Chryseobacterium* infection in rainbow trout, raised in flow-through raceways in India. By employing a combination of biochemical and molecular testing techniques, we successfully identified the bacterium. To assess its pathogenicity, we conducted virulence tests and a challenge assay. Furthermore, our research uncovered a distinctive strategy for controlling the growth of *Chryseobacterium* in vitro by determining its minimum inhibitory concentration against novel synthetic antimicrobial peptides and other antibiotics.

## Materials and methods

### Appropriate ethics declarations

The experimental protocols conducted and the use of rainbow trout in the study were approved by the Institute Animal Care and Use Committee (IACUC) of ICAR-Directorate of Coldwater Fisheries Research, Bhimtal India (File no. ICAR-DCFR/IACUC/12/06/2020/07-B) and were in accordance to the Institutional Biosafety Committee (IBSC), Department of Biotechnology (DBT) and Ministry of Science and Technology, Government of India under the Rules for “Manufacture, Use/Import/Export and Storage of Hazardous Microorganisms/ Genetically Engineered Organisms or Cells, 1989 (Rules 1989) of Environment (Protection) Act 1986.” The study was performed following the relevant guidelines and regulations. Moreover, we also affirmed that the methods and results presented in the study were in accordance with ARRIVE guidelines and regulations^32^.

### Sample collection

Twenty (n = 20) moribund juvenile rainbow trout (mean length 16.5 ± 0.125 cm; mean weight 34.5 ± 0.512 g) were obtained from three rainbow trout farms, located in the Indian Himalayan Region. Six diseased rainbow trout were sampled from the farm 1, while farm 2 and farm 3 provided 7 diseased individuals each. A representative sample of fish from the affected population in each farm was collected for diagnostic and research purposes. The occurrence of disease was not observed throughout the entire farm. Instead, the infections were confined to only 2–3 raceways within each farm. The collected diseased rainbow trout exhibited pathological manifestations, including haemorrhages on the dorsal body surface, black pigmentation, fin rot, and deep lesions in the caudal peduncle regions. In order to identify potential parasitic or viral infections, a subset of 10 moribund rainbow trout specimens was carefully selected for processing and examination, comprising 3 specimens from farm 1, 3 from farm 2, and 4 from farm 3. To achieve this, gill squash and body surface scrapings from the rainbow trout were collected on pre-cleaned slides and subjected to microscopic analysis to identify any parasitic infections. Additionally, degenerated PCR primers targeting the DNA polymerases of aquatic herpesviruses, poxviruses, iridoviruses, and adenoviruses in liver and kidney, were employed to screen for viral signatures^33,34^.

### Measurement of water quality parameters

Water samples were obtained from all the three rainbow trout farms for the assessment of crucial water quality parameters, including water temperature, dissolved oxygen, pH, total dissolved solids, and total ammonia nitrogen. Measurements were conducted using a digital probe (HANNA HI 9829, HI Media TDS/TEMP-3) and a commercial water quality parameters kit as per the manufacturer’s instruction (HIMEDIA WT028, WT042A).

### Isolation and growth conditions for *Chryseobacterium balustinum*

The remaining 10 live specimens were processed for the bacteriological investigation. Swab samples were collected from caudal peduncle regions of the infected trout specimens. For the collection of the tissue samples (liver and kidney), the trout juveniles were euthanized at 150 mg L^−1^ of Ethyl-3-aminobenzoate methanesulphonate (MS 222) to bring them to humane end points^35,36^. Then specimens were disinfected with 70% ethanol before collecting the tissue samples for isolation of pathogenic bacteria as etiological agents for the pathological conditions and infections recorded in the study. The tissue and swab samples were processed aseptically on different media; Shieh agar (supplemented with 0.5 µg mL^−1^ tobramycin; pH 7.2) and Hsu-Shotts agar (supplemented with 4.0 µg mL^−1^ neomycin sulphate; pH 7.2). The plates were incubated at 15 °C for 1 week and at 25 °C for 24–48 h. The plates incubated at 25 °C showed growth of yellow slimy pigmented colonies, whereas the plates incubated at 15 °C did not show the development of any yellow slimy pigmented colonies. The yellowish and slime-dominant colonies (n = 6) were selected randomly and screened for the 30% KOH test. The isolates were further sub-cultured on Tryptic Soya agar (TSA) at 25 °C for 24–48 h and preserved in 20% glycerol at − 20 °C for long-term storage.

### Biochemical analysis

The biochemical identification of the isolates was carried out as per the standard methods published earlier^37–39^. Following the manufacturer's protocol, the Gramme reaction of bacterial isolates was determined using a Gram staining kit (HI MEDIA K001-1KT). The isolates were tested for the basic biochemical reactions such as cytochrome oxidase, catalase, Simon citrate, methyl-red (MR), Voges-Proskauer’s reaction (VP), Hydrogen sulphide, starch and aesculin hydrolysis, DNase and gelatinase, urease, nitrate, indole, tyrosine, arginine dihydrolase, lysine decarboxylase and ornithine decarboxylase. They were also tested for the acid production from sugars; mannitol, raffinose, mannose, sucrose xylose, salicin, trehalose, inositol, glucose, arabinose and lactose for biochemical analysis. In brief, a piece of sterile filter paper impregnated with freshly prepared oxidase reagent was placed in a petri dish. A streak of test bacterial culture was smeared across the filter paper with the help of a sterile platinum loop cytochrome oxidase test. For the catalase test, the colony of the test bacterium was smeared onto a clean glass slide. Then, a drop of 30% H_2_O_2_ was placed on the smear. The ability of the test bacteria to utilize citrate as a sole source of carbon was tested by growing the culture on Simmons citrate agar slant for 24–48 h at 28 °C. For the MR test, a few drops of methyl red reagent were added to the 48–72 h grown culture in MR-VP broth. In the case of the VP test, VP reagents (solution A- 0.6 mL and solution B- 0.2 mL) were added to the 48–72 h grown bacterial culture in the MR-VP broth. Aseptically, a loopful of the bacterial culture was transferred to the Tryptic soy broth and incubated for 24–48 h at 28 °C. Once the bacterial growth was observed in the broth, ferrous sulfate solution as Hydrogen sulphide indicator was added to the broth grown with bacterial culture in Hydrogen sulphide test. Starch hydrolysis test was carried out by spot inoculating young test bacterial culture onto starch agar plate. After 18–24 h of incubation at 28 °C, the plates were flooded with Lugol’s iodine solution. In the case of the aesculin test, bacterial cells were inoculated into aesculin broth and incubated at 28 °C for 24–48 h. A loop of bacterial culture was streaked as a single line on DNase agar plates. The plates were incubated at 28 °C for 2–4 days. After the incubation period, the plates were flooded with 1% HCl. For the gelatinase test, the gelatin plate was spot inoculated with bacteria culture and incubated at 28 °C for 24–48 h. For the urease test, the test bacteria was allowed to grow on urea agar slant at 28 °C for 6–24 h and observed for 6 days. The nitrate test was conducted by stab or streak inoculation of the test culture onto nitrate agar tubes followed by incubating the tubes at 28 °C for 24–48 h. Then nitrate solution was added to the tube. The indole test was conducted by adding heavy inoculum of test culture into tryptone broth in tubes. The tubes were incubated for a period of 48 h at 28 °C. Then 6–7 drops of Kovacs reagent were added to the tubes. In the tyrosine test, a loopful test culture was inoculated into tyrosine broth and the culture was incubated at 28 °C for 24–48 h. After observing the bacterial growth, a Durham tube was inserted into the tyrosine broth and allowed to incubate at 28 °C for an additional 24–48 h. The ability of test bacterial culture to produce arginine dihydrolase, lysine decarboxylase and ornithine decarboxylase was tested by inoculating test culture in the medium containing these 3 amino acids in separate tubes. The tubes were overlaid with sterile liquid paraffin and incubated at 28 °C. Control tubes with the basal medium were also incubated for each test. The tubes were observed for a week. The bacterial abilities to produce acid from sugars; mannitol, raffinose, mannose, sucrose xylose, salicin, trehalose, inositol, glucose, arabinose and lactose were tested by supplementing the basal medium with individual sugar in separate tubes. Then tubes were inoculated with bacterial culture and incubated at 28 °C.

### Molecular characterization (16S rRNA gene) and phylogenetic analysis

Promega DNA isolation Kit was used to process the colonies that had grown on the agar plate in order to isolate the DNA and to analyse 16S rRNA gene by PCR. For PCR amplification universal primer UFF2 (5′-AGA GTT TGA TCC TGG CTC AG-3′) and URF2 (5′-ACG GGC GGT GTG TTC-3′) were used targeting the 16S rRNA gene. In the PCR reaction, the amplification of amplicons with a size of 1400 base pairs was achieved. The PCR product was sent for nucleotide sequencing by Sanger’s sequencing method with the help of an ABI Big Dye Terminator Cycle Sequencing kit v3.1 and ABI 3730 XL (Applied Biosystems). The partial forward and reverse sequences of the 16S rRNA gene were assembled (CLC Genomics Workbench software, version 11.0.1) and the similarity search was conducted with the other *Chryseobacterium* spp., using BLASTn (NCBI, Bethesda, MD, USA). The 16S rRNA genes of all the isolates (n = 6) were found 100% identical. The test isolate was designated as laboratory strain no RTFCP 298 and submitted to NCBI as *C. balustinum*, RTFCP 298 (OP 604186) as a representative. The closely related sequences of the *C. balustinum* RTFCP 298 and other species under the genus were retrieved from the NCBI along with type strains and some Indian isolates. The multiple alignments were done by CLUSTAL W^40^.

The aligned sequences were used to infer evolutionary history according to the neighbour-joining algorithm^41^ by Molecular Evolutionary Genetic Analysis (MEGA X)^42^. The Maximum Likelihood Method and Kimura 2-Parameter Model were used to infer the evolutionary history, and the tree with the highest log likelihood (− 6459.57) is presented, along with the percentage of associated taxa clustered together. To obtain the initial tree for the heuristic search, the Neighbour-Joining method was applied to a pair-wise distance matrix estimated using the Maximum Composite Likelihood (MCL) approach. The tree was drawn to scale, with branch lengths measured in substitutions per site. The analysis involved 22 nucleotide sequences, including 1st, 2nd, 3rd, and non-coding sites, with a total of 1548 positions in the final dataset. *Acinetobacter calcoaceticus* strain NCCB 22016 (NR042387) was included in the phylogenetic analysis as an out-group.

### Phenotypic determination of virulence characteristics

Biofilm assay^43^ and haemolytic activities were performed for phenotypic determination of the in vitro virulence characteristics of *C. balustinum* RTFCP 298.

### Biofilm assay

In 96-well flat bottom polystyrene microtiter plates, 30 µL of 10^8^ CFU mL^−1^ *C. balustinum* RTFCP 298 was seeded with 200 µL of different growth media (HSU- SHOT, Cytophaga and SHIEH) with glucose and without glucose (0.45%) and incubated at 25 °C for 24–48 h. The planktonic cells were removed by washing twice with phosphate buffer saline (PBS) at pH 7.4. The staining was done by adding 150 µL of 0.1% crystal violet and kept for incubation for 1 h at 25 °C followed by washing with PBS (pH 7.4). Finally, the stain acquired by adherent bacteria was resolved by adding 200 µL of 95% ethanol and kept at 4 °C for 1 h. The biofilm formation was quantified by measuring optical density at 590 nm.

### Haemolytic assay

The haemolytic assay was performed in 96 well U-bottom polystyrene microtitre plate, using defibrinated sheep blood^44^. The aseptically collected fresh defibrinated sheep blood was washed three times with PBS, centrifuged for 10 min at 1000×*g* and re-suspended at 10% (v/v) in PBS, containing 10 mM Dithiothreitol (DTT). The sheep erythrocytes were incubated with the freshly grown pure culture of *C. balustinum* RTFCP 298 in decreasing order of concentrations; 1.5 × 10^8^, 1.5 × 10^6^, 1.5 × 10^4^ and 1.5 × 10^2^ CFU mL^−1^ at 25 °C for 1 h. PBS (100 μL) and 0.2% Triton X-100 (100 μL) were used as a negative and positive control, respectively. The incubation of the plate was followed by centrifugation at 1000×*g* for 10 min and transferring of the supernatant to a flat bottom 96 well polystyrene microtitre plate. The absorbance of the plate was measured at 540 nm to estimate the lysis of the erythrocyte.

The percentage of haemolysis was calculated as:

Hemolysis (%) = 100 × [(A_Sample_ − A_PBS_)/(A_Triton X−100_ − APBS)], where 0.2% Triton X-100 and PBS are positive control and Negative control respectively.

### Resazurin assay for determination of minimum inhibitory concentration (MICs) of antibiotics and antimicrobial peptides (AMPs)

The minimum inhibitory concentrations (MICs) were measured in 96-well microtiter plates by broth dilution method^45^. The Muller Hinton broth medium (50 µL), containing decreasing concentrations (256–0.125 µg mL^−1^) of antibiotics (n = 5); erythromycin, florfenicol, neomycin, ampicillin and oxytetracycline and 15 AMPs, was inoculated with 10^5^ CFU mL^−1^ of *C. balustinum* RTFCP 298 in 96-well microtiter plates, with each plate containing a positive control (only bacterial cells) and negative control (broth without bacteria). The MICs were determined after 18–20 h of incubation at 25 °C by the lowest concentration of antibiotics and AMP at which no visible growth occurred. The 10 μL of freshly prepared Resazurin (0.015%) diluted in PBS was added to all the wells of the MIC plate and incubated at 25 °C for 2 h. The colour of the entire well was recorded. A blue colour in the well was interpreted as no growth, whereas the development of pink colour scored as growth of the test bacterium. The MIC was defined as the lowest antibiotic/peptide concentration, which prevents a colour change from blue to pink.

### Challenge assay

To assess the pathogenicity of *C. balustinum* RTFCP 298, we conducted an experimental infection in rainbow trout fingerlings having an average weight of 25.0 ± 0.028 g and a length 12.5 ± 0.50 cm. The healthy fingerlings were collected from the state rainbow trout farm at Bairangna in district Chamoli, Uttarakhand for the challenge assay. Prior to the experimental infection in a flow-through system, the test fish (n = 30) were acclimated to the wet laboratory settings for 15 days in FRP tanks, each with a capacity of 500 L. Gill, liver and kidney and swab samples collected from randomly selected rainbow trout fingerlings (n = 6) were processed on SHIEH and Hsu-Shotts agar media as described previously for the screening of *C. balustinum* before the commencement of experimental infection. The fresh culture of the test bacteria was incubated in SHIEH-broth at 25 °C for 24 h. The cells were pelleted and washed twice with sterile 0.85% PBS. For the experimental infection, we maintained a constant cell density of 3.0 × 10^6^, 3.0 × 10^7^ and 3.0 × 10^8^ CFU mL^−1^ (DEN-1 McFarland densitometer, Grant-bio, England). To test Koch's hypotheses, 100 µL of 3.0 × 10^6^, 10^7^ and 10^8^ CFU of *C. balustinum* RTFCP 298 was administered to healthy rainbow trout fingerlings intraperitoneally in triplicate treatment groups and monitored for disease progression, abnormal behaviour, feeding and mortality. The control group was injected with 100 µL of 0.85% phosphate buffer saline (PBS). The water temperatures of experimental tanks were maintained at 18 °C. Following the post-challenge phase, the infected fingerlings (n = 6) randomly selected from each experimental group were again processed aseptically on SHIEH agar medium supplemented with 0.5 g mL^−1^ tobramycin, Hsu-Shotts agar supplemented with 4.0 µg mL^−1^ neomycin sulphate and TSA, as described previously. This was done to re-isolate and confirm the presence of *C. balustinum* in the organs of the challenged rainbow trout. The experimental trial was conducted for 90 days.

### Histopathological analysis

The gill, liver, kidney, eye, spleen, intestine and muscle tissues from the experimentally infected rainbow trout were collected separately and fixed with Davidson’s fixative for 12–16 h^46,47^. The fixed tissue samples were gradually dehydrated and blocks were prepared in embedding ‘O’ ring (HI Media, India). In brief, the tissue samples were dehydrated in the ascending grades of ethyl alcohol (50–100%) and then soaked in the clearing agent xylene twice to remove the dehydrating agent completely. The tissues were embedded in melted paraffin wax at 60–61 °C for 4–6 h, followed by the preparation of the blocks in embedding ‘O’ ring. Thin sections (4.0 µm) were cut (Micron HM 325, Thermo Scientific, USA) and adhered to the double frosted glass slides using egg albumin and glycerol mixed in a ratio of 1:1. After baking the slides at 37 °C for 1 h, the tissue sections were stained with 2% H & E stain^48^ and further examined under the microscope (Leica DM 3000) for histopathological changes.

### Statistical analysis

Data on biofilm formation have been presented as mean ± standard error (Prism Version 5.01 GraphPad software). The significant differences (*p* < 0.01) in biofilm formation by *C. balustinum* RTFCPB 279 in different glucose-rich and glucose-limiting media were evaluated through One-way analysis of variance (ANOVA). To examine the significant variation (*p* < 0.01) in haemolytic activity between *C. balustinum* RTFCPB 279 concentration at 10^8^ and 10^6^ CFU mL^−1^, the paired ‘t’ test was performed. SPSS software version 19.0 (SPSS Inc, Chicago IL) was used for the statistical analysis in the present study.

## Results

### Water quality parameters

During the collection of diseased rainbow trout samples, the average values of water quality parameters in the trout farms were as follows: water temperature of 18.5 °C, the dissolved oxygen concentration of 8.5 mg L^−1^, pH of 7.5, total dissolved solids of 45 mg L^−1^, and total ammonia nitrogen (TAN) level of 0.01 mg L^−1^, indicating that the parameters were within the optimum range.

### Clinical signs and bacterial isolation

The clinical signs, recorded in the study, were black pigmentation, haemorrhage in the dorsal body surface, fin rots and deep lesions at caudal peduncle regions. The microscopic examination of samples, prepared from diseased rainbow trout, did not reveal the presence of any parasitic infections. The PCR-based detection for aquatic herpesviruses, poxviruses, iridoviruses and adenoviruses using degenerated primers yielded negative results. The circular yellow colonies (n = 6) appeared on HSU-SHOT agar medium after 24–48 h of incubation at 25 °C. The pigmentation test (30% KOH reaction) detected the presence of flexirubin agent in all 6 isolates by turning yellow, slime bacterial colonies to brick red in colour.

### Biochemical analysis

The isolates were positive for Gram’s reaction, catalase and cytochrome oxidase test. The other basic biochemical reactions showed that the isolates were negative for DNase, gelatinase, methyl red, VP reaction, Simon citrate and starch hydrolysis. They produced acid from mannitol, raffinose, mannose, sucrose, and xylose. The details of biochemical results are given in Table 1.

**Table 1:** Biochemical characteristics of *C. balustinum* RTFCPB 279 isolated from diseased rainbow trout.

Tests	Result	Tests	Result
Flexirubin pigment	( +)	Xylose	( +)
Hemolysis	( +)	D- Galactose	( +)
Gliding motility	( +)	D- Arabinose	(−)
Growth at 4 °C	( +)	D ( +) Melibiose	(−)
Growth at 30 °C	( +)	Maltose	( +)
Urease production	( +)	D- Raffinose	( +)
Citrate assimilation	(−)	D ( +) Mannose	( +)
Cytochrome oxidase	( +)	Sucrose	( +)
Catalase	( +)	Mannitol	(−)
Methyl red	(−)	VP reactions	(−)
Nitrate reduction	( +)	DNase	(−)
Indole	(−)	Starch hydrolysis	(−)
Arginine dihydrolase	(−)	Ornithine decarboxylase	(−)
Lysine decarboxylase	(−)	Tyrosine	(−)
Trypsine	( +)	Hydrogen sulphide	(−)
Aesculin hydrolysis	( +)	Gelatinase	(−)

‘+’: positive; ‘−’: negative

### Molecular characterization and phylogenetic analysis of *C. balustinum*, RTFCP 298

**Figure 1:**
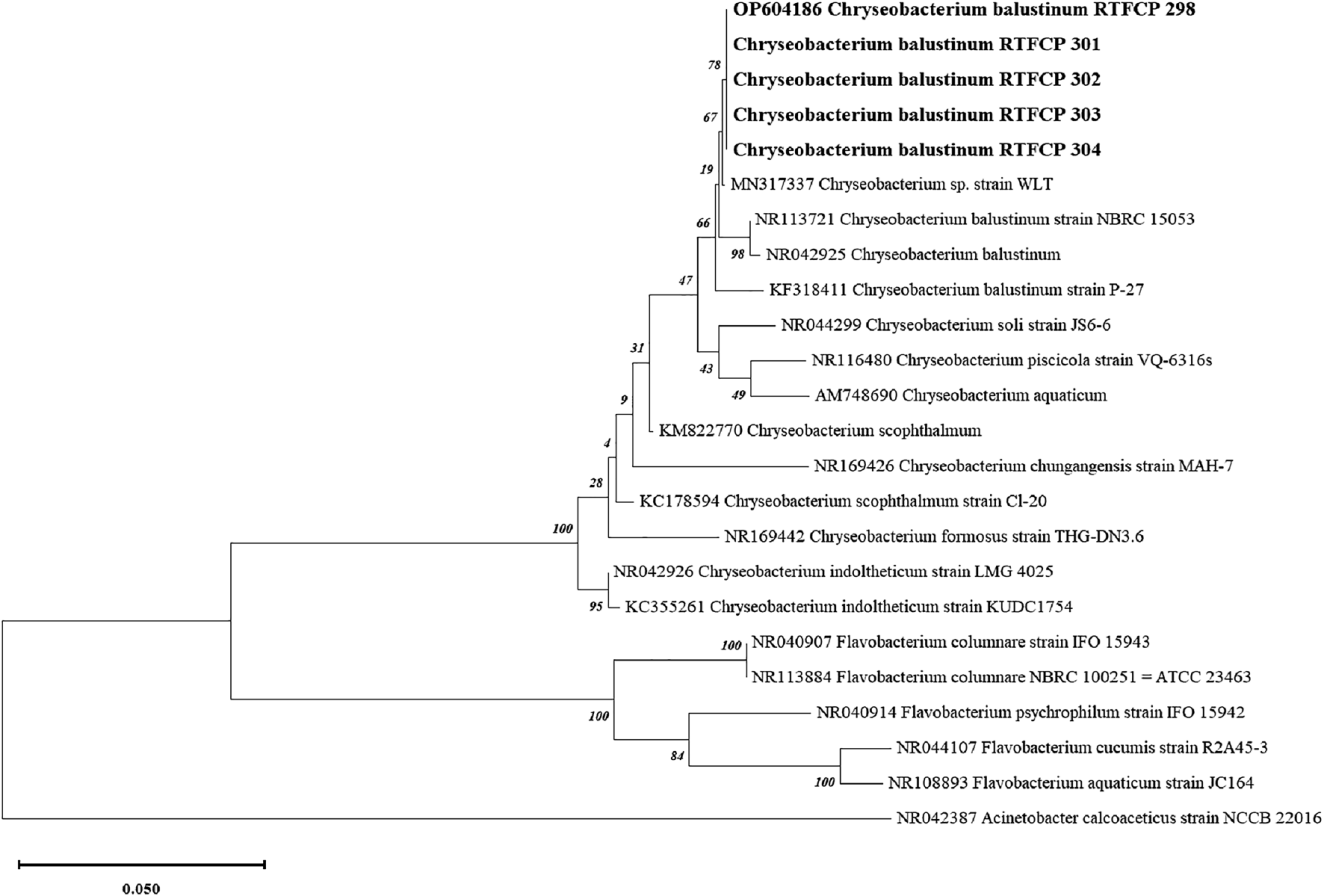
Phylogenetic analysis was conducted using the maximum likelihood method with the kimura-2-parameter model, employing 500 bootstrap replications that included the 1st, 2nd, 3rd, and non-coding sites. The resulting tree with the highest log likelihood (− 6449.11) is presented, indicating the percentage of trees where associated taxa clustered together alongside the branches. Initial trees for the heuristic search were generated by applying the Neighbour-Joining method to a pair-wise distance matrix estimated using the Maximum Composite Likelihood (MCL) approach. The tree is scaled, with branch lengths measured in substitutions per site. This analysis involved 24 nucleotide sequences, and the final dataset consisted of 1541 positions. All evolutionary analyses were performed using MEGA X.

The molecular characterization (16S rRNA gene) and phylogenetic analysis of the sequences of *C. balustinum*, RTFCP 298 shared the maximum similarity of 99.85% with *C. balustinum* strain WLT (MN317337) followed by 99.77% with *C. balustinum* P-27 (KF318411), 99.33% with *C. balustinum* (NR 042925) and 99.15% with *C. balustinum* NBRC 15053 (NR 113721). *C. balustinum* strains RTFCP 301, 302, 303, and 304 exhibited 100% sequence similarity with *C. balustinum* RTFCP 298, suggesting a close genetic relationship among these isolates. These strains were recovered alongside *C. balustinum* RTFCP 298 from the diseased rainbow trout samples as described in the “Materials and Methods” section. The phylogenetic tree involved analysis of 24 nucleotide sequences, and also established the identification of the present strain RTFCP 298 as *C. balustinum* in the study (Fig. 1).

### MICs determination

MICs were determined against 5 different antibiotics and 15 synthetic novel antimicrobial peptides. All the tested antimicrobials showed a varying pattern of antimicrobial activity against *C. balustinum* RTFCP 298, where the MICs varied from 1 to > 256 Μg mL^−1^ against antibiotics; oxytetracycline, erythromycin, florfenicol, neomycin and ampicillin (Table 2). Similarly, the MICs of synthetic antimicrobial peptides varied from 8 to > 256 Μg mL^−1^ (Table 3).

**Table 2:** Minimum Inhibitory Concentration of antibiotics against *C. balustinum* RTFCP 298.

S. No	Antibiotic	Minimum Inhibitory Concentration (Μg mL^−1^)
1	Erythromycin	2
2	Florfenicol	16
3	Neomycin	4
4	Ampicillin	> 256
5	Oxytetracycline	1

**Table 3:** Antimicrobial activity of synthetic peptides against *C. balustinum* RTFCP 298.

S. No	Peptide	Minimum inhibitory concentration (Μg mL^−1^)
1	P1 (CNQ-20)	> 256
2	P2 (LF-10)	64
3	P3 (RY-10)	> 256
4	P4 (LL-20)	256
5	P5	256
6	P6	> 256
7	P7	32
8	P8 (FV-20)	256
9	P9 (IL-20)	16
10	P10 (RR-20)	8
11	P11	64
12	P12	32
13	P13	> 256
14	P14	16
15	P15	> 256

### Virulence characteristics

#### Biofilm

**Figure 2:**
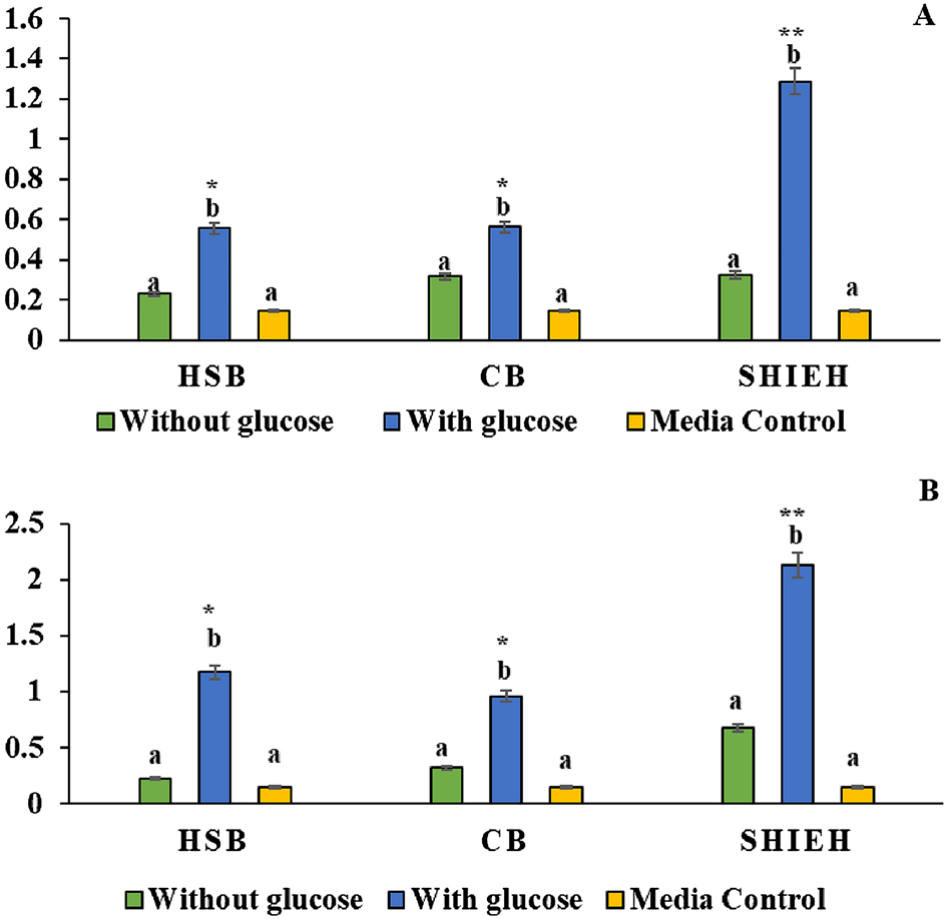
(**A**–**B)** Biofilm formation by *C. balustinum* RTFCP 298 in glucose-rich and glucose-limiting medium at (**A**) 24 h and (**B**) 48 h; HSB: HSU-Shot broth, SHIEH: Shieh broth, and CB: Cytophaga broth. Significant variation in biofilm formation recorded between glucose-rich and glucose limiting conditions with the medium (*p* < 0.01). The biofilm formation was also significant in glucose rich SHIEH medium at 24 h and 48 h (*p* < 0.01) as compared to other glucose-rich media.

The biofilm formation test revealed that in the presence of glucose, *C. balustinum* RTFCP 298 formed significant biofilm (*p* < 0.01) as compared to glucose-limiting and medium control within the medium over the experimental period (24–48 h). When compared between glucose rich-media; HSB, CB and SHIEH, the biofilm formation was significant in glucose rich SHIEH medium at 24 h and 48 h (p < 0.01) (Fig. 2A-B).

#### Haemolytic assay

**Figure 3:**
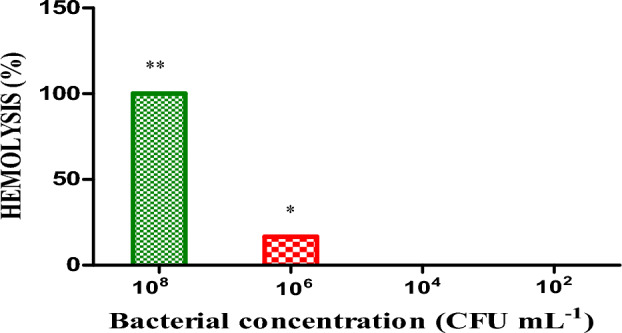
Phenotypic expression of haemolytic activity of *C. balustinum* RTFCP 298. Haemolysis (%) calculated against +ve control Triton X-100 (0.2%) and Neat 10^8^ CFU mL^−1^.

*C. balustinum* RTFCP 298 exhibited haemolytic activity on sheep erythrocytes. At 1 × 10^8^ CFU mL^−1^ of the test culture, 100% haemolysis was observed, while at a two-fold serial dilution (10^8^ CFU mL^−1^), the haemolytic activity was 16.6%, confirming the virulence characteristic of *C. balustinum* RTFCP 298 in vitro. Further dilutions in the concentration of *C. balustinum* RTFCP 298 did not yield any haemolytic activity (Fig. 3).

### Challenge study

**Figure 4:**
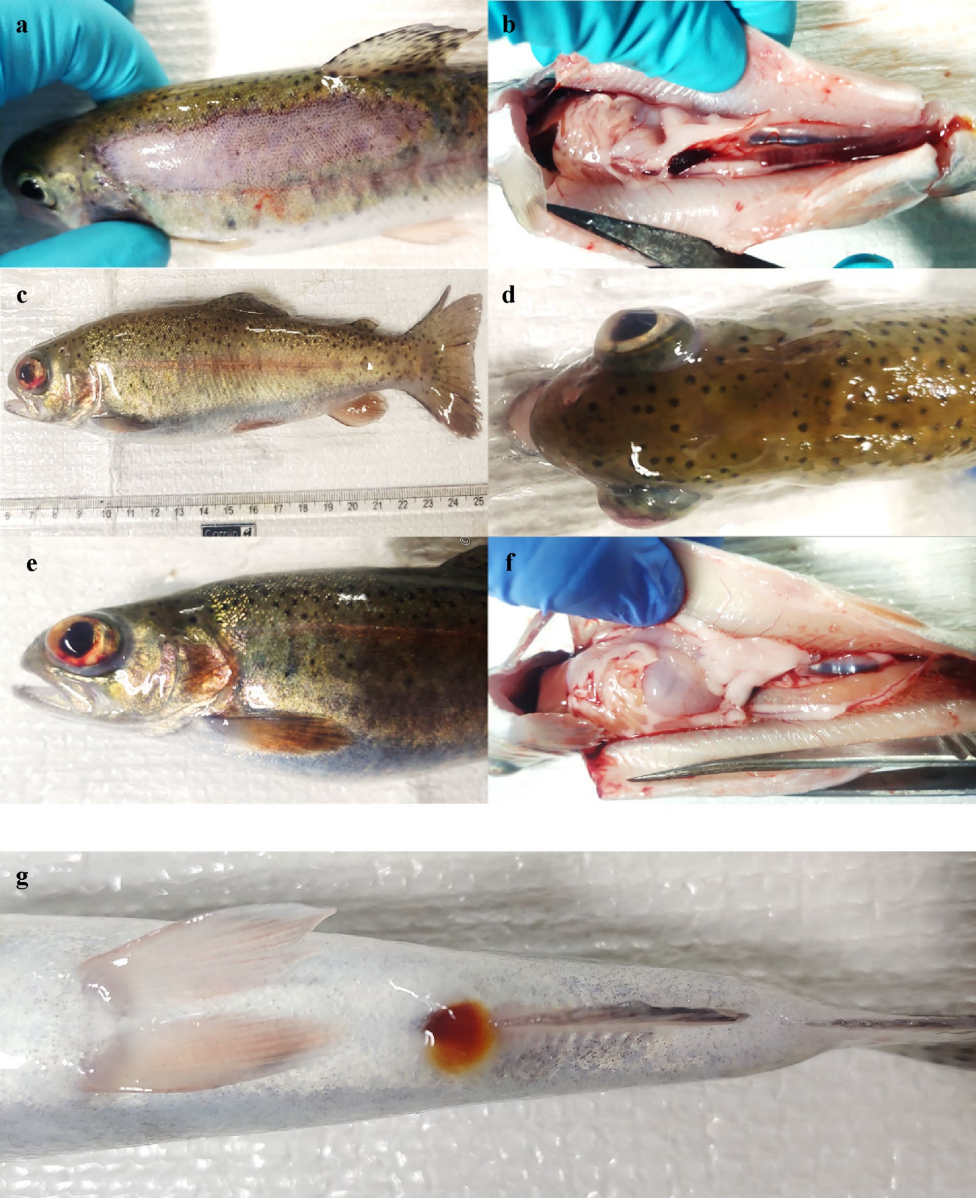
Gross clinical and pathological signs in rainbow trout, *O. mykiss* experimentally challenged with *C. balustinum.* (**a**) Dermal necrosis or diffuse lateral scale-pocket. (**b**) Sub-peritoneal region- splenomegaly and diffuse petechiae. (**c**) Eye haemorrhage in the anterior chamber. (**d**) Exophthalmia, haemorrhages in the eye and dorsal discoloration are visible in the cranial region. (**e**) Eye haemorrhage in the anterior chamber (f) haemorrhaging and liquefaction in internal organ and (**g**) ascites discharge from anus.

*C. balustinum* was found moderately pathogenic to rainbow trout fingerlings in the experimental infection to confirm Koch's postulate. The post-challenge period progressed with the development of initial pathological conditions and disease signs such as haemorrhage in eyes, black pigmentation, red patches and lethargy on the second day in all the experimental groups. During the entire course of experimental duration (90 days), the development of skin rashes and skin erosion at the base of the pectoral fins and the marked reduction in feed intake capacity among the challenged fish were recorded. Haemorrhage in the eyes of experimentally infected fish gradually turned into unilateral and bilateral exophthalmia conditions also. The necropsy of defunct or recently dead experimental fish revealed liquefaction in internal organs, haemorrhage in the liver and abnormal gall bladder (Fig. 4).

### Mortality

First mortality episodes of 12% and 15% were recorded in the fish group challenged with 3.0 × 10^7^ and 3.0 × 10^8^ CFU mL^−1^of *C. balustinum* RTFCP 298, respectively post 4th day of the experimental infection. The experimental group challenged with 3 × 10^6^ CFU mL^−1^ of the test bacterium did not cause any mortality, but showed development of few signs of disease progression. On the 15th day of the post-challenge period, 4% mortality was recorded in the fish group infected with 3 × 10^6^ CFU mL^−1^ of *C. balustinum* RTFCP 298. LD_50_ value of *C. balustinum* RTFCP 298 was recorded at 10^8^ CFU mL^−1^, which led to the 50% mortality of the test fish on the 29th day. The fish in the control group of the experiment displayed no signs of disease progression, and no mortality was observed among the fish in this group. *C. balustinum* RTFCP 298 was re-isolated from the infected rainbow trout on SHIEH agar medium supplemented with 0.5 g mL^−1^ tobramycin and Hsu-Shotts medium supplemented with 4.0 g mL^−1^ neomycin sulphate, and phenotypic and partial 16S rRNA gene homology confirmed the characterization. A few isolates of *Aeromonas* group were also identified on TSA plate in the study.

### Histopathology

**Figure 5:**
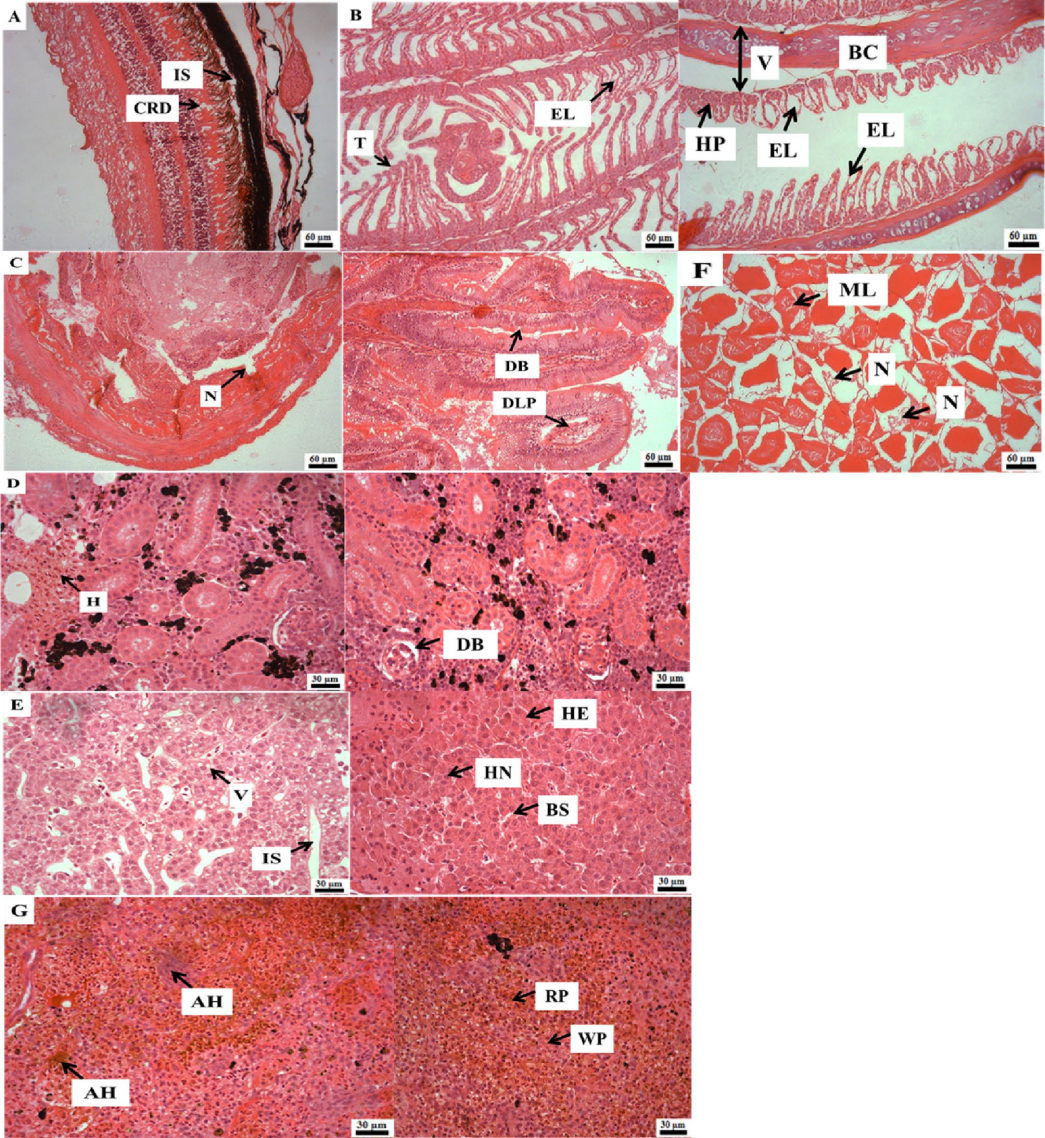
Photomicrograph of histological sections (4 μm) of rainbow trout challenged with *C. balustinum* RTFCP 298. (**A**) Eye: Increase space (IS) between pigmented epithelium and photoreceptor layer and Cone-rod dystrophy (CRD); (**B**) Gill: Epithelial lifting (EL), Telangiectasia (T), Blood congestion (BC), Hyperplasia (HP) and Vasodilatation (V); (**C**) Intestine: Necrosis (N), disruption of brush border (DB), Degeneration of lamina propria (DLP); (**D**) Kidney: Haemorrhage (H), Dilation of Bowman's space (DB); (**E**) Liver: Vacuolation (V), Increase of sinusoidal space (IS), Hepatocyte (HE), Hepatocyte nucleus (HN), Blood sinusoids (BS); (**F**) Muscle: Necrosis (N), Myocyte losses (ML), (**G**) Spleen: Accumulation of hemosiderin (AH), White pulp (WP), Red pulp (RP).

Histopathological alterations in gill tissue included fusion of primary and secondary gill lamellae, hypertrophy, hyperplasia, vacuolation, epithelial lifting, telangiectasia in secondary lamellae, blood congestion and vasodilatation. The liver showed vacuolation, increasing sinusoidal space, hepatocyte nucleus and blood sinusoids, whereas haemorrhage and dilation of Bowman's space were recorded in the kidney. Spleen showed accumulation of hemosiderin, white and red pulp. The eye had increased space between pigmented epithelium and photoreceptor layer and cone-rod dystrophy. Necrosis, disruption of brush border and degeneration of lamina propria were observed in the intestine, whereas muscle showed necrosis and myocyte losses (Fig. 5).

## Discussion

In the present study, *C. balustinum* RTFCP 298 isolates were recovered and characterised from the natural infection of rainbow trout in the Indian Himalayan Regions (IHR). The disease signs were similar to the cases of infection of *Flavobacterium* spp. in fish^49^. Since the first isolation in halibut, it has been recognised as a pathogenic bacterium rather than food spoilage bacterium, because it was isolated from the fin^4,26,50^.

*C. balustinum* RTFCP 298 was identified based on the colony morphology, physiological and biochemical characteristics^20^. It was further confirmed by the PCR amplification of the 16S rRNA gene and phylogenetic analysis. The 16S rRNA gene is the most commonly used gene marker for identifying bacteria up to the species level. The 16S rRNA gene sequence alignment of *C. balustinum*, RTFCP 298 and phylogenetic analysis revealed that it shared maximum sequence similarity with *C. balustinum* strain WLT (MN317337)^20^. It is proposed that 16S rRNA gene identification can be useful in diagnosing *C. balustinum* infection in fish. *Chryseobacterium* WLT (MN 317337) from rainbow trout in the Republic of Korea revealed 99.24% similarity to other *Chryseobacterium* group^20^. Similarly, 16S rRNA gene of *C. scophthalmum* isolated from golden mahseer TPBLGL 18 (KM822770) has 99% similarity to the 16S rRNA gene of *C. scophthalmum* strain LMG 13028T (NR 025386)^24^.

Microdilution test utilizing resazurin revealed the lowest minimum inhibitory concentration against *C. balustinum* strain for oxytetracycline, florfenicol, and erythromycin. Due to their wide-ranging effectiveness and reduced harm to fish, the fish farmers in India frequently employ oxytetracycline, tetracycline, and ampicillin to combat bacterial diseases^51^. Consequently, the utilization of any of these three antibiotics could serve as a potential control measure during *C. balustinum* infection in trout. Previous studies have documented the natural resistance of *Chryseobacterium* spp. to a subset of antibiotics, including tetracyclines, erythromycin, linezolid, polymyxins, and chloramphenicol^52^. Furthermore, in our investigation, we assessed the antimicrobial efficacy of 15 synthetic novel antimicrobial peptides (AMPs) against *C. balustinum*. Remarkably, 5 out of the 15 AMPs exhibited notable antimicrobial activity (8–32 Μg mL^−1^) against the target bacterium. The susceptibility of peptides to bacterial interference may be attributed to their short length, which increases the likelihood of possessing unique sequences. As only a limited number of drugs and chemicals are approved for use in the treatment of diseases in aquaculture, an epizootic might cause significant losses in fish farming operations during the outbreak^53^.

The molecular virulence mechanism of non-model organisms such as *C. balustinum* can be better understood using information acquired from model species' virulence mechanisms. Few of the pathogenic determinants of *C. balustinum* have been investigated in vitro by comparative research on virulent colony types such as haemolytic test and biofilm formation in the current study. *C. balustinum's* haemolytic activity, like that of *F. columnare*, has been observed to have higher expression and detection against the potent haemolytic agent Triton X-100 (0.2%)^36^. Hemolysin of other organisms was identified by its ability to lyse RBCs or affect the biological functioning of other cells^54^. Proteases can play an important role in the formation of biofilm, which determines pathogenicity in bacteria^55,56^. The biofilm formation test revealed that *C. balustinum* was able to form biofilm with or without glucose in the medium. The biofilm generating bacteria are thought to be the primary factor for many opportunistic infections in fish, and they are extremely difficult to eliminate due to their much-enhanced resistance (1000 times) to several antimicrobials. As a result, greater concentrations of antimicrobial drugs must be used to kill or restrict the formation of pathogenic microbial consortiums established in biofilms^57^.

Several studies from India reported *F. branchiophilum* and *F. columnare* infections in farmed Indian major carps (IMCs), ornamental fishes and rainbow trout^36,58–60^. Visible clinical symptoms such as gill lesions and cutaneous haemorrhage and ascites within the peritoneum, among other symptoms, have also been recorded in golden mahseer infected with *Flavobacterium* species^4,39,59,61,62^. In our study, *C. balustinum* RTFCP 298 was found to be moderately pathogenic to rainbow trout juveniles in the experimental infection trial to confirm the Koch's postulate test by revealing symptoms like dermal necrosis, liquefication, sub-peritoneal region- splenomegaly and diffuse petechiae, eye haemorrhage in the anterior chamber, internal organ haemorrhaging and ascites discharge from anus. Though the challenge test was conducted for 90 days, the pathogenicity of *Chryseobacterium* RTFCP 298 at experimental doses of 10^6^, 10^7^ and 10^8^ CFU mL^−1^ caused moderate mortality in rainbow trout. In general, *Chryseobacterium* pathogenicity in farmed fish is lower than that of the major bacterial pathogens (e.g., *Flavobacterium* spp and *Lactococcus garvieae*). Although the pathogenicity of *Chryseobacterium* is not much higher than that of other common pathogens in aquaculture, a 15-d experimental infection trial in rainbow trout weighing 20 g on average by *Chryseobacterium* strain WLT at 3 × 10^7^ CFU of bacterium demonstrated 60% mortality and the LD_50_ of the same strain recorded in between 10^6^ to 10^7^ CFU of the bacterium (Jung et al.^20^). This Indian isolate, *Chryseobacterium* RTFCP 298 has shown its LD_50_ at 10^8^ CFU mL^−1^. In the present study, the reproduction of clinical signs of disease progression and isolation of *C. balustinum* in the organs of the challenged rainbow trout established the test of Koch's hypotheses. However, detection of a few *Aeromonas* isolates on TSA plates may be correlated with the possible stress induction in such a long challenge test.

The present study investigated the histopathological changes associated with experimental infections of *C. balustinum* in rainbow trout. The gill tissue exhibited various alterations including necrotic deformities, fusion of primary and secondary gill lamellae, hypertrophy, hyperplasia, and vacuolation. Gill bacterial disease in fish is characterized by lamellar fusion and lamellar epithelial cell hyperplasia, as previously reported^63^. Similar histological changes in gill tissue have been observed in salmonid^4^, golden mahseer^24^ and young turbot^64^ infected with *C. scophthalmum* and other *Chryseobacterium* sp. Additionally, other organs such as the liver, eye, kidney, and spleen exhibited pathological changes such as vacuolation, increase of sinusoidal space, hepatocyte and hepatocyte nucleus alteration, blood sinusoid changes, increased space between pigmented epithelium and photoreceptor layer, cone-rod dystrophy, haemorrhage, dilation of Bowman's space, accumulation of hemosiderin, white pulp, and red pulp. Therefore, clinical signs and histopathological alterations suggested that the presence of disease in rainbow trout was due to the *C. balustinum* infection.

## Conclusion

The present study reports the susceptibility of the test isolate, *C. balustinum*, to a range of FDA-approved antibiotics commonly used in aquaculture. Notably, this is the first report of the isolation of *C. balustinum* in the Indian context. The present findings underscore the potential of appropriate antibiotic treatment to effectively manage *C. balustinum* infection in farmed trout. Importantly, this research contributes to the identification and control of the disease in rainbow trout farming and highlights the necessity of implementing a regular monitoring and inspection regime. Therefore, analysis of infected samples from trout farms could aid in the prevention of *C. balustinum* and other diseases from spreading in aquaculture production units.

## Data Availability

The nucleotide sequence (16S rRNA) of *Chryseobacterium balustinum* RTFCP 298 is submitted to the Genebank NCBI and available to the public with accession number OP604185. All the data generated in the study, are present in this article.
